# A systematic review and meta-analysis of clinical efficacy of early and late rehabilitation interventions for ischemic stroke

**DOI:** 10.1186/s12883-024-03565-8

**Published:** 2024-03-08

**Authors:** Xufang Wei, Shengtong Sun, Manyu Zhang, Zhenqiang Zhao

**Affiliations:** 1 Hainan Medical University International School of Public Health and One Health, Haikou, Hainan China; 2 Department of Neurology, First Affiliated Hospital, Hainan Medical University, Haikou, Hainan China

**Keywords:** Ischemic stroke, Rehabilitation, Meta-analysis, Randomized controlled trial

## Abstract

**Introduction:**

At present, stroke has become the first cause of death and disability among Chinese adults. With the coming of the aging population in China, the disease burden brought by stroke will be increasingly aggravated. And stroke is a leading cause of disability. There is a golden plastic period after stroke, during which timely and safe intervention and rehabilitation therapy can effectively improve the disability status. However, there is still controversy about the duration of interventional rehabilitation after stroke. This study conducted a meta-analysis on the influence of intervention in early and late ischemic stroke rehabilitation.

**Method:**

Chinese language databases such as CNKI, Wanfang, and VIP, and English language databases such as Embase, PubMed, Web of Science, and The Cochrane Library were searched, and RCT related to early and late rehabilitation of ischemic stroke from the establishment of the database to October 2023 was collected. Review Manager 5.4.1 was used for relevant analysis. The main outcomes were Barthel Index or Modified Barthel Index, Fugl-Meyer Assessment scale, NIHSS, China Stroke Scale. Standardized Mean Difference (SMD) was used as an effective indicator of continuity variables, and the estimated interval was expressed by 95% confidence interval (CI).

**Results:**

A total of 1908 patients were included in 16 studies. The results showed that, compared with late rehabilitation, early rehabilitation improved clinical efficacy. Barthel Index or Modified Barthel Index score was [SMD = 1.40, 95%CI(1.16,1.63), *p* < 0.001]; the score of Fugl-Meyer Assessment Scale was [SMD = 1.18, 95%Cl (0.85, 1.52), *P* < 0.001]; the score of NIHSS was [SMD= -0.44, 95% CI(-0.65, -0.24), *P* < 0.001]; the result of China Stroke Scale score was [SMD= -0.37, 95%CI(-0.56, -0.18), *P* < 0.001].

**Conclusion:**

In comparison with late rehabilitation, early rehabilitation can significantly improve self-care abilities, daily activities, and neurological functions of ischemic stroke patients.

**Trial registration:**

This meta-analysis has been registered with Prospero, and the registration number is CRD42022309911. The registration period is March 22, 2022.

## Introduction

Stroke includes ischemic stroke and hemorrhagic stroke, and more than 80% is due to ischemic stroke [1]. Ischemic stroke is often accompanied by different degrees of nerve damage and dysfunction, among which limb motor dysfunction is the most common [2]. Limb motor dysfunction after ischemic stroke is often manifested as limb weakness, poor joint flexion and extension, and complete immobility of limbs, which poses a serious threat to patients’ normal life and brings a heavy burden to family and society [3].

Post-stroke rehabilitations are commonly used to help the stroke patients to regain their abilities in their daily lives [4]. Studies have shown that three months after ischemic stroke is the golden period of nerve remodeling, and rehabilitation can significantly promote functional recoveries of the stroke patients [5]. However, at present, timing of the most effective rehabilitation (i.e. early vs. late) are still controversial [6]. Some studies have shown that early rehabilitation has a positive effect, improving patients’ ability to perform activities of daily living [7, 8]; Some studies have shown that early rehabilitation has potential hazards compared with late rehabilitation, such as associated complications [9, 10]. Whether early rehabilitation can improve the prognosis and quality of life of stroke patients remains to be verified.Therefore, the purpose of this study was to conduct a meta-analysis on the effects of early vs. late interventional rehabilitations on functional recoveries in patients with ischemic strokes, to provide evidence for best practice for interventional rehabilitations after ischemic stroke.

## Methods

### Inclusion and exclusion criteria of literature

#### Type of research

Randomized Controlled Trial (RCT).

#### Human objects of study

Inclusion criteria: (1) Diagnostic criteria were based on either Chinese Diagnostic Guidelines for Acute Ischemic Stroke 2010 or the diagnostic criteria of stroke by the Fourth National Conference on Cerebrovascular Diseases, and confirmed by head CT or MR imaging; (2) The patient’s vital signs and nervous system were stable; (3) The patient agreed to participate in rehabilitation training.

Exclusion criteria: (1) patients with either severe lung infection, liver disease, kidney disease, heart disease or other vital organ damage; (2) patients suffering from severe cognitive impairments and unable to receive rehabilitation training.

#### Intervention measures

Experimental groups: early rehabilitation (i.e., starting within 2 weeks post ischemic stroke); Control group: late rehabilitation (i.e., starting after 2 weeks post ischemic stroke).

#### Outcome indicators

(1) Modified Barthel Index (MBI) or Barthel Index (BI); (2) Fugl-Meyer Assessment Scale (FMA); (3) National Institute of Health Stroke Scale (NIHSS); (4) China Stroke Scale(CSS).

#### Exclusion criteria

(1) Non-RCT study; (2) Repeated publication; (3) No full text or incomplete data; (4) Inconsistent rehabilitation time, or no clear specific rehabilitation time point; (5) No corresponding outcome index.

### Literature search strategy

Chinese language databases, such as CNKI, Wanfang and VIP, and English language databases, such as PubMed, Web of Science, and The Cochrane Library were searched to collect RCT data related to early and late ischemic stroke rehabilitation from the establishment of the databases up to October 2023. More specifically, English language search terms include: “ischemic stroke,” “cerebral infarction,” “cerebral embolism,” “early rehabilitation,” “late rehabilitation,” and “acute rehabilitation”; and Chinese language search terms included: “ischemic stroke”, “cerebral infarction”, “cerebral embolism”, “cerebrovascular disease”, “rehabilitation”, “rehabilitation nursing”, “acute stage”, “early stage”, “late stage”, “different time”, and “different timing”. Taking PubMed as an example, the specific retrieval strategy is as follows:

- #1 Search: ischemic stroke
- #2 Search: cerebral infarction
- #3 Search: cerebral embolism
- #4 Search:#1OR#2OR#3
- #5 Search: early rehabilitation
- #6 Search: late rehabilitation
- #7 Search: acute rehabilitation
- #8 Search: #5OR#6 OR #7
- #9 Search: #4AND#8
- #10 Search: #4AND #8 Filters: Randomized Controlled Trial

### Literature screening and data extraction

The data were extracted separately by two independent researchers and cross-checked, and differences between the two independent searches were resolved through discussion and negotiation. In the literature screening process, titles and abstracts of literatures were screened first, and literatures that were clearly irrelevant were excluded; full texts that passed the initial screening were screened investigated further to determine whether or not those studies should be included. Data extracted from the included studies were title, author, publication time, design type, allocation method, age, case in the early rehabilitation, case in the late rehabilitation, rehabilitation starting time, outcome assessment scale, efficacy evaluation time, and outcome indicators.

### Risk assessment of bias in the included studies

The Cochrane bias risk assessment tool was used to assess the risk of bias in the included studies. After the independent assessment by two independent researchers, cross-checks were carried out, and any disagreement was resolved through consultation and reference to relevant data.

### Risk of bias across studies

We assessed the possibility of publication bias by evaluating funnel plots of the trials’ mean differences for asymmetry. Publication bias was found to be small.

### Statistical analysis

Review Manager 5.4.1 was used for analysis. As the literatures included in this study belong to clinical intervention studies, there are large heterogeneity, such as the age of the study subjects, the severity of the lesions, and the intervention measures of rehabilitation treatment, etc. so the random effects model is selected for literature analysis. Standardized Mean Differences (SMD) was selected as the effective index of the continuity variable, and the estimated interval was expressed by 95% confidence interval (CI).

## Results

### Literature screening process and results

**Fig. 1:**
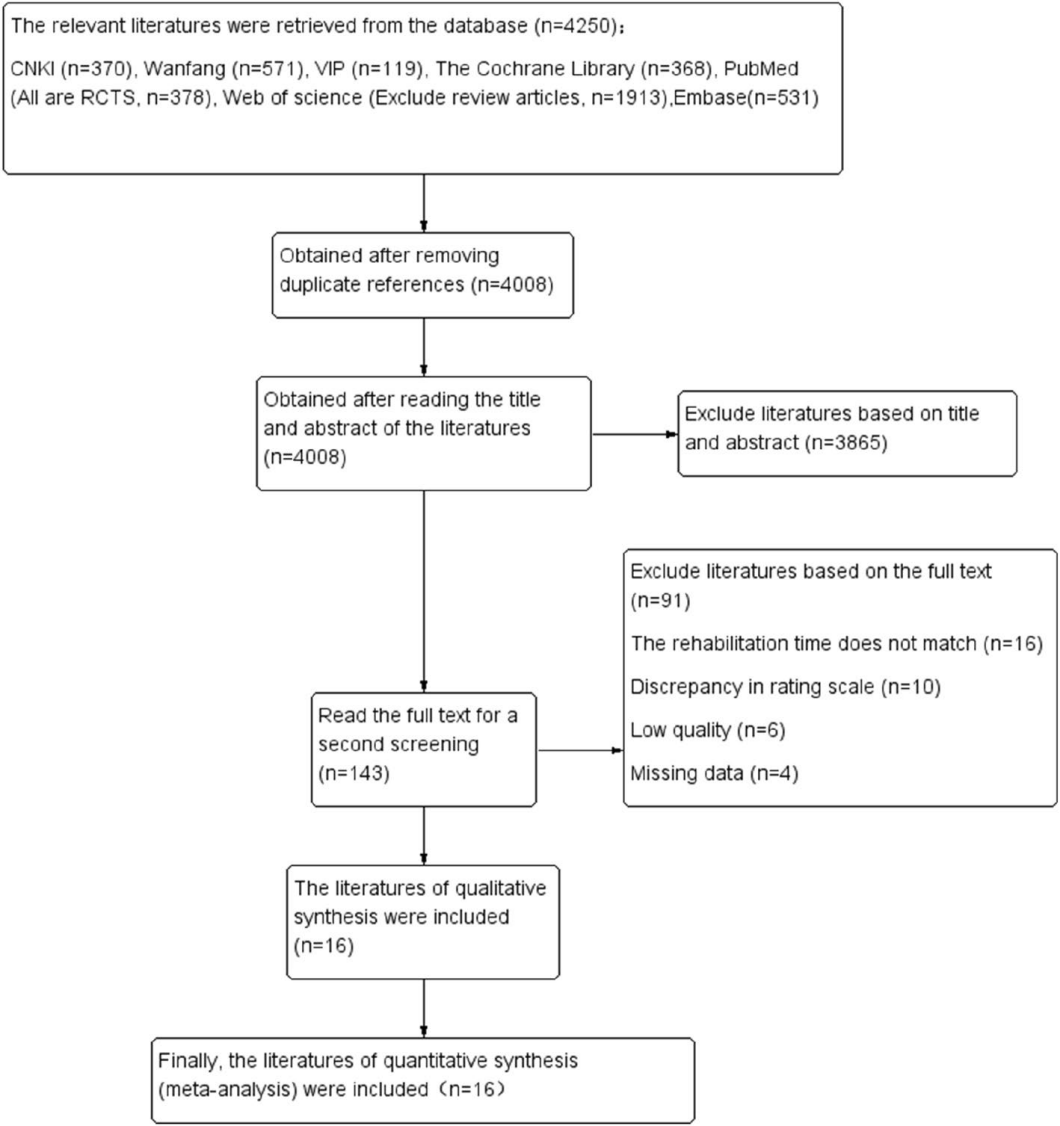
Literature screening process and results from each step of the process

A total of 4250 literatures were obtained in preliminary search, and 16 RCTs were eventually included after layer-by-layer screening [11–26]. Of the patients in the included studies, there were 1908 patients with ischemic stroke 959 patients received early rehabilitation and 949 patients received late rehabilitation. The literature screening process and results are shown in Fig. 1.

### Basic features included in the study

The basic features of the included research are shown in Table 1. Table 1 is at the end of the manuscript.

**Table 1:** General characteristics

Reference	Subjects number		Age(years)		Start time of rehabilitation		Time of assessment	Indicators of evaluation
	Experimental group	Control group	Experimental group	Control group	Experimental group	Control group		
Wang Jingru 2002 [11]	55	45	64.1	64.2	between one and five days	Day fourteen	1 month	②
Zhang, D. J. 2005 [12]	108	108	57.91 ± 11.35	58.55 ± 10.49	Between two and fourteen days	Between Fifteen and thirty days	1 month	②③⑤
Chen Li 2005 [13]	46	46	49.3 ± 12.4	49.8 ± 12.6	Between one and three days	After fourteen days	1 month	①③
Chen Mingyuan 2006 [14]	108	108	40–75	40–75	In fourteendays	After fourteen days	1 month	②③
Zhu Jianling 2006 [15]	53	52	60.8 ± 7.6	59.4 ± 8.2	After two days	After fifteen days	1 month	②③⑧
Peng Kun 2015 [16]	50	50	42–74	42–74	Between two and fourteen days	Between fifteen and thirty days	1 month	②③
Liu Fahua 2016 [17]	30	30	59.2 ± 1.2	59.2 ± 1.2	In seven days	After fourteen days	2 weeks, 4 weeks	①③
Bian Xin 2016 [18]	47	51	63.12 ± 7.84	61.32 ± 9.46	In fourteendays	Between fifteen and thirty days	1month, 3 months	②③④
Liu Fen 2017 [19]	63	63	56.9 ± 3.78	56.7 ± 3.24	Between two and fourteen days	Between fifteen and thirty days	1 month	②③
Zhang Shuhong 2022 [20]	90	90	71.48 ± 3.70	71.50 ± 3.69	In fourteendays	Between fourteen and Twenty-eight days	four weeks	①③④⑦
Li Yongping 2017 [21]	39	36	59.67 ± 16.89	58.41 ± 15.57	In two days	After fourteen days	3months	①③④
Zong Junxue 2004 [22]	108	108	57.91 ± 11.35	58.55 ± 10.49	Between two and fourteen days	Between fifteen and thirty days	1month	②
Liu Jiangbo 2018 [23]	40	40	59.5 ± 1.8	59.5 ± 1.8	Between one and fourteen days	Between fourteen and twenty-eight days	3months	②
Liu Weixia 2016 [24]	30	30	61.2 ± 1.6	61.3 ± 1.7	Between one and fourteen days	Between fourteen and twenty-eight days	3months	②③
Yu Jie 2009 [25]	54	54	67.6	65.7	Between one and five days	After fourteen days	1month	②③
Xu Baoshan 2023 [26]	38	38	58.78 ± 5.33	58.84 ± 5.26	In seven days	After twenty-one days	1month	①③

Notes: Experimental group = Early rehabilitation; Control group = Late rehabilitation

①Barthel Index (BI), ②Modified Barthel Index (MBI), ③Fugl-Meyer Assessment Scale (FMA), ④National Institute of Health Stroke Scale(NIHSS), ⑤ China Stroke Scale (CSS), ⑥Modified Rankin Scale (MRS), ⑦Inflammatory Factors, ⑧Mini-Mental State Examination (MMSE)

### Evaluation of literature quality

**Fig. 2:**
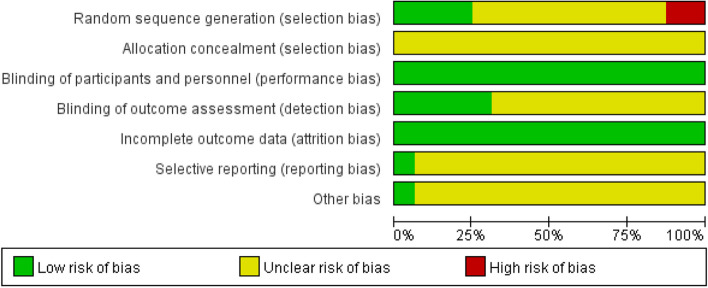
Risk of bias graph

**Fig. 3:**
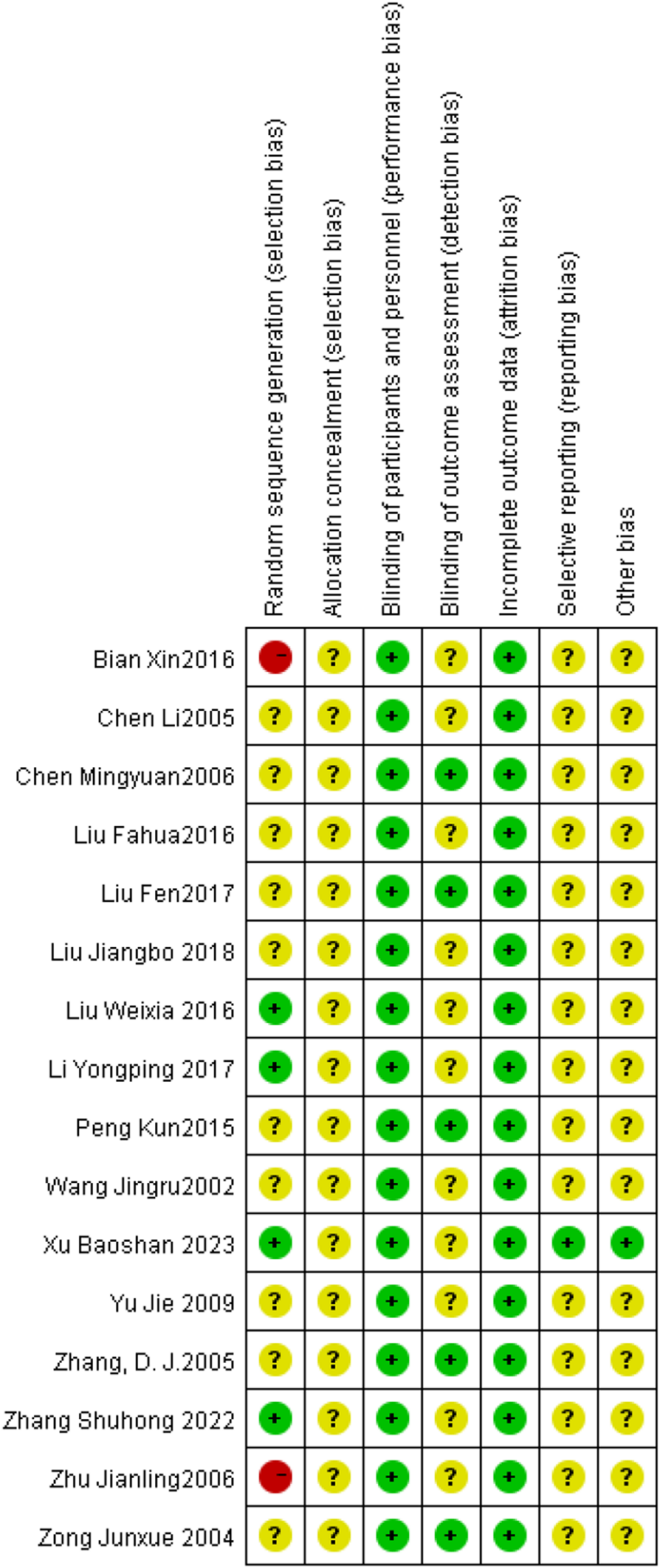
Risk of bias summary

Each randomized controlled trial will be evaluated in Review Manager 5.4.1. The results of bias risk assessment are shown in Figs. 2 and 3.

### Results of meta-analysis

#### Barthel Index or Modified Barthel Index

**Fig. 4:**
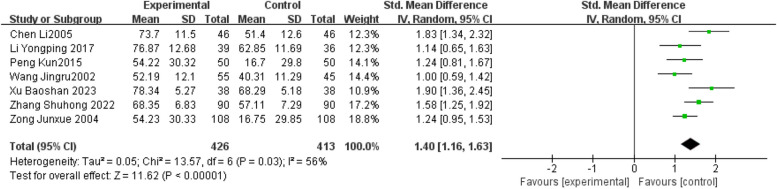
Meta-analysis results of Barthel Index or Modified Barthel Index

Sixteen studies used Barthel Index or Modified Barthel Index [11–26]. The combined analysis of Barthel Index and Modified Barthel Index evaluation data showed clear heterogeneity among the studies. Through the sensitivity analysis of each study, it was found that nine studies [12, 14, 15, 17–19, 23–25] had clear heterogeneity, possibly due to the different ages and intervention measures of the research objects. Therefore, the nine studies were excluded. Finally, 7 studies [11, 13, 16, 20–22, 26] were included for combined analysis. There are 426 cases in the early rehabilitation and 413 cases in the late rehabilitation. The heterogeneity of the included references in this study was large, and random effects model was selected for analysis. The results showed that early rehabilitation training was more effective than late rehabilitation to improve motor function in patients with ischemic strokes (SMD = 1.40, 95%CI [1.16,1.63], and the difference was significant (z = 11.62, *p* < 0.00001). The results of each study are shown in Fig. 4.

#### Fugl-Meyer Assessment scale

**Fig. 5:**
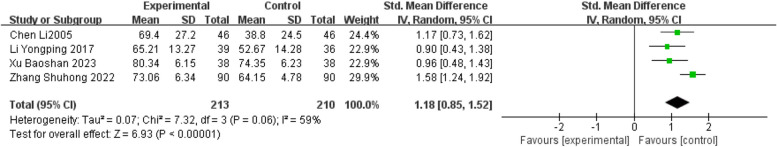
Meta-analysis results of Fugl-Meyer Assessment scale

Fugl-Meyer Assessment scale was included in 13 studies. However, only 7 [13, 16–18, 20, 21, 26] studies were included for analysis, and the other 6 [12, 14, 15, 19, 24, 25] were excluded because Fugl-Meyer Assessment scale for upper and lower limbs were not combined. Through the sensitivity analysis of each included study, it was found that 3 studies [16,17,18,]had relatively larger heterogeneity. This was likely due to larger age groups and intervention measures the study employed; therefore, the study was excluded. As a result, 4 [13, 20, 21, 26] studies were included. In total, three are 213 cases in the early rehabilitation and 210 cases in the late rehabilitation. The heterogeneity of the included studies in this study was large, and random effects model was selected for analysis. The final combined result was SMD = 1.18, 95% CI[0.85, 1.52], and the difference was significant (z = 6.93, *p* < 0.00001 ). The results showed that the early rehabilitation group had a higher Fugl-Meyer Assessment score than the late rehabilitation group, suggesting that early rehabilitation post ischemic stroke was more effective than late rehabilitation for motor functional recovery. The results of each study are shown in Fig. 5.

#### NIHSS

**Fig. 6:**
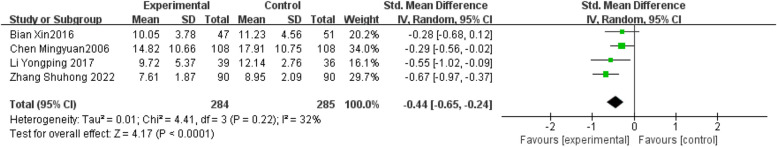
Meta-analysis results of NIHSS

NIHSS Assessment scale was included in 4 studies [14, 18, 20, 21]. Three are 284 cases in the early rehabilitation and 285 cases in the late rehabilitation. The heterogeneity of the included studies in this study was large, and random effect model was selected for analysis. The final combined result was SMD=-0.44, 95%CI [-0.65, -0.24], and the difference was significant (z = 4.17, *p* < 0.0001). The results showed that the NIHSS of the early rehabilitation group was lower than that of the late rehabilitation group, suggesting that the early rehabilitation was more effective to enhance functional recovery after ischemic stroke than late rehabilitation. The results of the studies are shown in Fig. 6.

#### China stroke scale

**Fig. 7:**
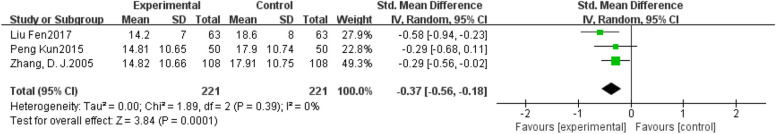
Meta-analysis results of China Stroke Scale

Three [12, 16, 19] included studies employed neurological impairment scale using China Stroke Scale. In the three studies, there were 221 cases in the early rehabilitation and 221 in the late rehabilitation. The heterogeneity of the included studies in this study was large, and random effects model was selected for analysis. The final combined result was SMD= -0.37, 95%CI [-0.56, -0.18], and the difference was significant (z = 3.84, *p* = 0.0001). The results showed that the neurological impairment scale in the early rehabilitation group was lower than that in the late rehabilitation group, indicating that the neurological function recovery in the early rehabilitation of ischemic stroke was more effective than that in the late rehabilitation. The results of each study are shown in Fig. 7.

## Discussion

This meta-analysis showed that early rehabilitation can improve neurological function and quality of life in ischemic stroke patients compared with late rehabilitation, which is mainly manifested by significant improvements in NIHSS score, CSS score, BI/MBI score and FMA score. Through systematic review, we confirmed for the first time the positive role of early rehabilitation within two weeks in the recovery of neurological function and the improvement of quality of life in patients with ischemic stroke, which provided a certain theoretical basis for the application and promotion of early rehabilitation of stroke.

Prior to this systematic review, we understood that the evidence linking timing of rehabilitation (i.e., early vs. late) to stroke recovery was inconsistent in the literature. Relevant studies have shown that very early and more frequent mobilization after stroke does not affect patients’ quality of life [27–29]. In fact, studies even suggest that that very early and high dose mobilization is harmful to patients’ rehabilitation [30, 31]. This may be related to very early initiation of rehabilitation combined with high doses of rehabilitation training. This finding provides evidence against too early intervention in rehabilitation. Our results suggest that early rehabilitation within 2 weeks after stroke is beneficial to neurological function and quality of life, compared with late rehabilitation, which starts 2 weeks after stroke. This may be because early rehabilitation prevents some of the musculoskeletal, cardiovascular, respiratory, and immune systems-related effects of bed rest [32]. On the other hand, it may be due to the increased sense of self-efficacy that brings patients greater confidence. Relevant studies also show that early activity is related to the increase of Barthel index [33], which is consistent with the results of Barthel index analysis in this study.

However, this meta-analysis also has some limitations. In terms of mobilization for rehabilitation at the very early stage (i.e., within the first 48 h), this study cannot provide a reference basis, because the starting time of rehabilitation in the literature included in this study is within 2 weeks. In addition, fewer studies were included, and differences in intervention protocols were inevitable. Due to the inherent heterogeneities associated with strokes, such as stroke intervention measures, and dosages there are needs for better and more systematic studies on the efficacy of rehabilitation post stroke. Therefore, it is hoped that there will be more multi-centered high-quality RCTs in the future to discuss related issues of early stroke rehabilitation to better determine the optimal time and dosage of early rehabilitation.

## Conclusions

Compared with late rehabilitation, early rehabilitation (starting within 2 weeks post ischemic stroke) can significantly improve the self-care ability, daily activities, and neurological function of ischemic stroke patients. However, this study does not provide evidence for intervention in very early (less than 48 h after stroke onset) rehabilitation.

## Data Availability

Datasets are available through the corresponding author, upon reasonable request.

## Abbreviations

CNKI: China national knowledge infrastructure
RCT: Randomized Controlled Trial
MD: Mean Difference
CI: Confidence interval
MBI: Modified Barthel Index
BI: Barthel Index
FMA: Fugl-Meyer Assessment Scale
NIHSS: National Institute of Health Stroke Scale
CSS: China Stroke Scale
